# Differential Electrographic Signatures Generated by Mechanistically-Diverse Seizurogenic Compounds in the Larval Zebrafish Brain

**DOI:** 10.1523/ENEURO.0337-21.2022

**Published:** 2022-03-28

**Authors:** Joseph Pinion, Callum Walsh, Marc Goodfellow, Andrew D. Randall, Charles R. Tyler, Matthew J. Winter

**Affiliations:** 1 Biosciences, College of Life and Environmental Sciences, University of Exeter, Exeter, Devon EX4 4QD, United Kingdom; 2 Medical School, University of Exeter, Exeter, Devon EX4 4PS, United Kingdom; 3 Living Systems Institute and Mathematics, College of Engineering, Mathematics and Physical Sciences, University of Exeter, Exeter, Devon EX4 4QF, United Kingdom

**Keywords:** 3Rs, drug discovery, electrophysiology, neuropharmacology, seizures, zebrafish

## Abstract

We assessed similarities and differences in the electrographic signatures of local field potentials (LFPs) evoked by different pharmacological agents in zebrafish larvae. We then compared and contrasted these characteristics with what is known from electrophysiological studies of seizures and epilepsy in mammals, including humans. Ultimately, our aim was to phenotype neurophysiological features of drug-induced seizures in larval zebrafish for expanding knowledge on the translational potential of this valuable alternative to mammalian models. LFPs were recorded from the midbrain of 4-d-old zebrafish larvae exposed to a pharmacologically diverse panel of seizurogenic compounds, and the outputs of these recordings were assessed using frequency domain analysis. This included analysis of changes occurring within various spectral frequency bands of relevance to mammalian CNS circuit pathophysiology. From these analyses, there were clear differences in the frequency spectra of drug-exposed LFPs, relative to controls, many of which shared notable similarities with the signatures exhibited by mammalian CNS circuits. These similarities included the presence of specific frequency components comparable to those observed in mammalian studies of seizures and epilepsy. Collectively, the data presented provide important information to support the value of larval zebrafish as an alternative model for the study of seizures and epilepsy. These data also provide further insight into the electrophysiological characteristics of seizures generated in nonmammalian species by the action of neuroactive drugs.

## Significance Statement

In this study, we offer novel insight into the frequency domain of the local field potentials (LFPs) for a range of seizurogenic compounds in zebrafish larvae. We make a direct comparison of seizurogenic compounds with varying mechanisms of action and, where possible, compare the effects of these compounds in zebrafish larvae with those recorded in mammals in terms of the frequency components of their LFPs. This study adds to the mounting body of evidence supporting the use of the larval zebrafish as a powerful alternative model organism for seizure and epilepsy research.

## Introduction

Seizures are defined as periods of excessive or hypersynchronous brain activity (Fisher et al., 2014a), which, when recurrent and unprovoked, define the chronic disease epilepsy (Falco-Walter et al., 2018). In addition, seizure occurrences and myoclonus appear to be a feature of the pathophysiology of a number of other CNS diseases, including Alzheimer’s disease and other forms of dementia (Beagle et al., 2017). Seizures themselves have a wide-ranging etiology that includes a substantial proportion attributable to the adverse action of drugs. It has been estimated, for example, that ∼6% of new-onset seizures and 9% of status epilepticus cases are as a result of drug toxicity (Chen et al., 2016), which includes the inadvertent action of multiple marketed drugs (Ruffmann et al., 2006; Easter et al., 2009). Seizures themselves can be definitively identified in human patients and nonclinical animal models using neurophysiological assessment techniques such as local field potential (LFP) recordings and electroencephalography (EEG; Lévesque and Avoli, 2019; Usman et al., 2019). Using these techniques, seizures can be observed to present diverse electrographic dynamics, with some common components including low-voltage fast activity, e.g., high-frequency oscillations (HFOs; Jiménez-Jiménez et al., 2015; Wang et al., 2020) in the fast ripple frequency bands (250–500 Hz), or high-amplitude periodic spikes (Perucca et al., 2014; Jiménez-Jiménez et al., 2015; Wang et al., 2020). However, precisely how, and where, they are manifest can vary between different forms of epilepsy and different causes of seizure (Perucca et al., 2014; Jiménez-Jiménez et al., 2015; Wang et al., 2020).

In order to better understand why seizures occur and how to treat epilepsy, the use of experimental models is important. Clearly such models should include an appropriate, often complex neural architecture, and the best models are those performed *in vivo*, preferably in the absence of confounds generated by anesthesia. With this in mind, a drive toward more ethical and cost-effective approaches for studying complex neurologic disorders has increased interest in alternative, nonmammalian, vertebrate models for such studies. Of these models, the larval zebrafish offers considerable potential as a highly genetically tractable alternative for screening epilepsy related genes and for the study of genetic mutation-induced spontaneous seizures (Baraban et al., 2013; Griffin et al., 2021). To date, zebrafish have been relatively widely used for assessing drug-induced neural activity, and various studies have described the effects of seizurogenic chemicals on their behavior (Berghmans et al., 2007; Winter et al., 2008; Alfaro et al., 2011; Afrikanova et al., 2013; Baraban et al., 2013), electrophysiology (Baraban et al., 2007; Hortopan et al., 2010; Kim et al., 2010b; Afrikanova et al., 2013; Baraban et al., 2013; Cho et al., 2017; Griffin et al., 2017; Hunyadi et al., 2017; Copmans et al., 2019; Liu and Baraban, 2019), and functional imaging phenotypes (Winter et al., 2017; Burgstaller et al., 2019; Ghannad-Rezaie et al., 2019; Burrows et al., 2020; Winter et al., 2021). Electrophysiological assessments, in particular, offer extremely high temporal resolution and allow direct comparison of zebrafish-derived data with that obtained from neurophysiological assessments undertaken in mammals. Consequently, the recording of larval zebrafish electrophysiological data offers the opportunity to directly compare abnormal electrographic dynamics between these two taxonomically diverse sets of model organisms. In larval zebrafish, typically, LFP recordings from small clusters of neurons in easily identified anatomic targets (such as the optic tectum) are used to measure the response of the brain to drug treatment. Indeed, LFP recordings have been used to assess the electrographic response of the larval zebrafish to a few seizurogenic compounds including picrotoxin, pilocarpine (Hortopan et al., 2010; Baraban, 2013), and, most often, pentylenetetrazole (PTZ; Baraban et al., 2007; Kim et al., 2010b; Copmans et al., 2019; Liu and Baraban, 2019). Despite the relatively widespread use of electrophysiology in larval zebrafish, little is actually known about the specific electrographic characteristics of seizures induced by diverse chemicals in this model organism, other than the aforementioned exceptions. There are few published studies which characterize LFP profiles in larval zebrafish across a range of excitatory mechanisms, for example, or that have compared their characteristics with those used to define seizures in more traditional models of seizures and epilepsy using mammalian electrophysiology and human EEG data. Here, we sought to address this knowledge gap by assessing the LFP-based response of 4-d postfertilization (dpf) larval zebrafish, to treatment with a variety of seizurogenic compounds which act through a range of pharmacological mechanisms of action. Using frequency domain analysis on the resulting data we aimed to assess the similarities and differences in the electrographic signatures of LFPs between different pharmacologies, and to compare and contrast these characteristics with what is known from electrophysiological studies of seizures and epilepsy in mammals including humans, thereby adding considerably to our knowledge of the translational value of this model.

## Materials and Methods

**Figure 1.**
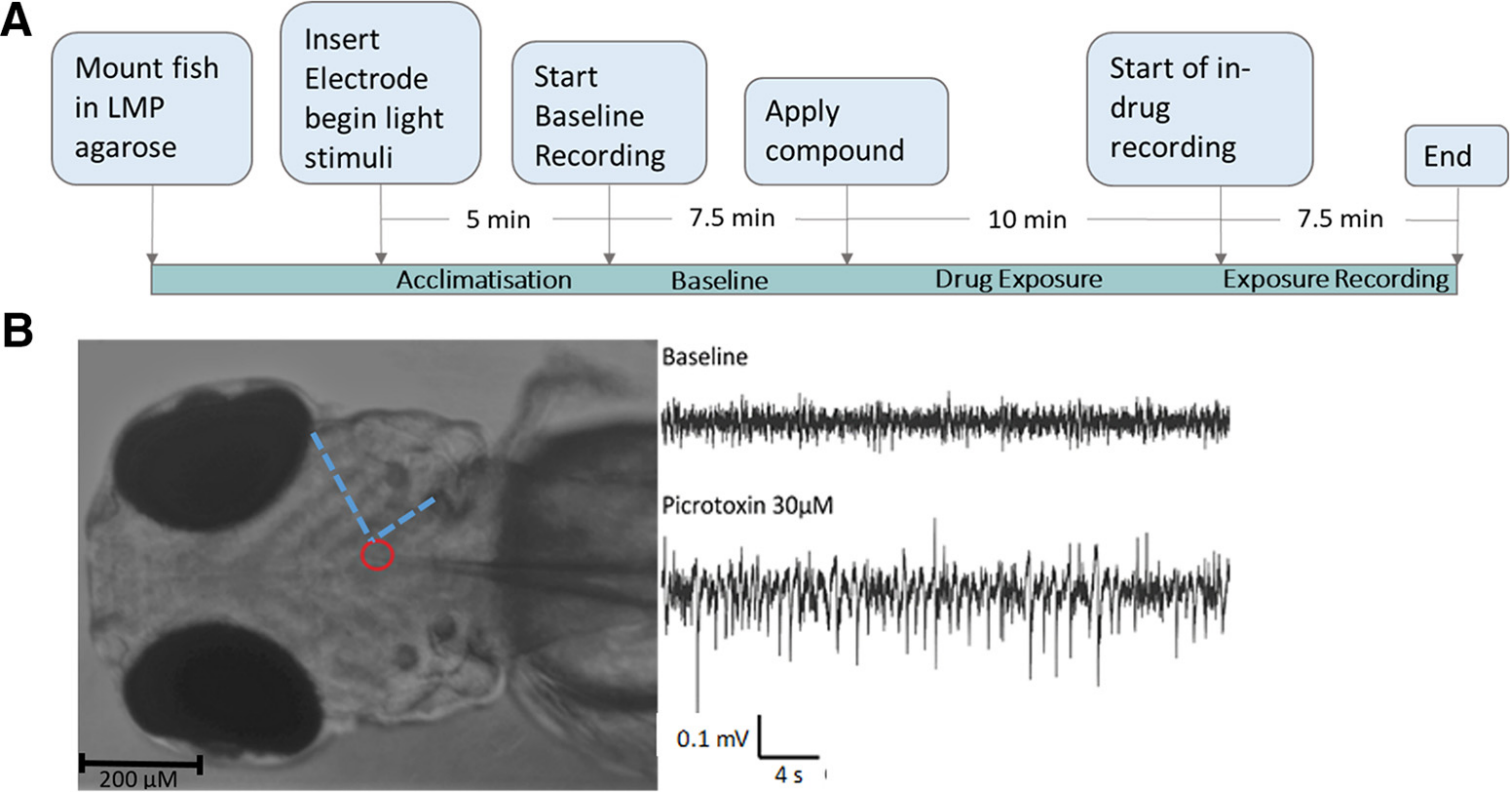
A schematic showing the experimental process and example data from *in vivo* electrophysiological recording in 4-d post fertilisation *elavl3:GCaMP6s* zebrafish larvae. ***A***, The experimental process used. ***B***, Left, A paralyzed, mounted zebrafish larva with glass electrode inserted into midbrain. The Red circle indicates the placement of the tip of the electrode, while the blue dashed line indicates landmarks used to consistently place the electrode. Right, local field potential recording (LFP) from midbrain of zebrafish larva at baseline and after administration of 30 μm picrotoxin.

The experimental approach used is detailed in the following sections and the process is summarized in Figure 1*A*. Throughout, the data shown in graphs and tables are the means ± SEM of data points from individual animals (*n*). All analyses were performed using MATLAB (MATLAB version 9.8.0, 2020), including the signal processing toolbox 8.4 (MATLAB signal processing toolbox 8.4, 2020) and wavelet toolbox 5.4 (MATLAB wavelet toolbox 5.4, 2020).

### Experimental animals

For this work, 4-dpf transgenic zebrafish, of unknown sex, with a pan-neuronal Ca^2+^ sensor (*elavl3*:GCaMP6s) were used. This transgenic line allowed us to compare the electrographic recording data generated here, with functional imaging data obtained from a separate study (Winter et al., 2021). Four-day postfertilization larval zebrafish were selected as these are not considered protected vertebrates under European animal legislation and as such are ethically preferable to the use of older, protected, animals. The use of 4-dpf larval zebrafish also enabled the use of neuromuscular blocking agents and for electrophysiological recordings to be performed in the absence of a potentially confounding general anesthetic.

Adult *elavl3:*GCaMP6s broodstock (Vladimirov et al., 2014; kindly supplied by Dr. Misha Ahrens, Janelia Research Campus, Howard Hughes Medical Institute, Ashburn, VA) were held in aquaria at 28 ± 1°C under optimal conditions for spawning. Briefly, animals were held in dechlorinated mains tap water (referred to here as “culture water”), which was routinely monitored for water quality parameters. Fertilized eggs were collected shortly after spawning and transferred to Petri dishes which were filled with culture water and held at 28 ± 1°C until use in experiments at 4 dpf (for full details, see Winter et al., 2017). All work was undertaken under project and personal licences granted by the United Kingdom Home Office under the United Kingdom Animals (Scientific Procedures) Act.

### Test compounds and concentration range selection

Test compounds were selected based on their known seizurogenic potential in mammals, including humans, as defined by case studies of seizure incidence and assays in model organisms as outlined in Table 1. Appropriate exposure concentration ranges ensuring CNS responsiveness without generalized toxicity were also selected based on previously published data (Winter et al., 2021). The exposure conditions adopted are summarized in Table 1. All compounds were dissolved in extracellular solution (ECS) and pH adjusted to ∼7.5 before use. The composition of ECS was: 1 mm NaCl, 2.9 mm KCl, 10 mm HEPES, 1.2 mm MgCl_2_, 10 mm dextrose, and 2.1 mm CaCl_2_ (Baraban, 2013). For each compound, there were three exposure concentrations, and for each experimental group, seven to eight larvae were used per treatment group. All test chemicals and other reagents were obtained from Sigma-Aldrich or Tocris.

**Table 1:** Test compounds and exposure concentration ranges used for *in vivo* electrophysiological recording in 4-dpf *elavl3*:GCaMP6s zebrafish larvae

Compound name	Pharmacodynamics	Concentrations used	Seizure liability
Aminophylline	Adenosine receptor antagonist and phosphodiesterase inhibitor	1, 2, 4 mm	Various cases of seizure in humans, and evidence of kindling in rats (Schwartz and Scott, 1974; Albertson et al., 1983)
Chlorpromazine	Dopamine, serotonin, histamine, Muscarinic and α1- and α2-adrenergic receptor antagonist	31.25, 62.5, 125 μm	Increased risk of seizure in patients receiving anti-psychotic drug (APD) treatment, particularly polytherapy; seizure risk 5-fold higher in individuals receiving low/medium potency APDS such as chlorpromazine (Bloechliger et al., 2015; Druschky et al., 2019)
Donepezil	Acetylcholinesterase inhibitor	125, 62, 31 μm	Among the top 10 drugs most commonly associated with seizures in World Health Organization adverse drug reaction database (Kumlien and Lundberg, 2010)
Picrotoxin	GABA_A_ receptor antagonist	30, 60, 120 μm	Commonly used convulsant compound used for modelling seizures (Mackenzie et al., 2002)
(RS)-(tetrazol-5-yl)glycine	NMDA receptor agonist	62.5, 125, 250 μm	Convulsant compound used for modelling seizures (Schoepp et al., 1991)
SB205607 (TAN-67)	δ-Opioid receptor agonist	125, 250, 500 μm	Increases incidence of convulsions in bicuculline kindled rats (Yajima et al., 2000)

Also shown are published evidence to support seizurogenicity. All compounds were sourced from Sigma-Aldrich or Tocris.

### *In vivo* LFP recordings from zebrafish brains

Individual 4-dpf larvae were paralyzed using 4 mm tubocurarine and then positioned dorsal side up in the recording chamber in 1.5% low melting point agarose containing ECS. Throughout recording, the mounted fish were kept in a static bath containing ECS. Under 4× magnification, a glass 2 m NaCl filled microelectrode (resistance of 3–5 MΩ) was inserted into the midbrain to record extracellular LFPs from a small network of neurons (Fig. 1*B*). The area chosen for electrode placement was based on clear anatomic landmarks, and the electrode was placed a small distance (∼30 μm) lateral from the midline of the brain. This was decided on to ensure consistency of electrode placement in a specific brain area. Recordings were captured using an Axon CNS Multiclamp 700B amplifier and digitized using an Axon Digidata 1440A (Amplifier settings were Mode: I = 0, Gain: 20, Bessel: 2 kHz), and data were recorded with the Clampex 10.4 software. Following introduction of the recording electrode, larvae were equilibrated for 300 s before beginning data acquisition. The LFP recording procedure consisted of acquiring 7.5 min of baseline data following which the test compound was added to the ECS by pipette, followed by recording in the presence of compound for 17.5 min. The exposure epoch was defined as the period between 17.5 and 25 min, thus allowing 10 min for the test compound to penetrate the larva and reach equilibrium. All raw electrophysiological signals were recorded at 10 kHz, downsampled to 2 kHz and digitally filtered, using a Butterworth filter, with high pass at 1 Hz and low pass at 500 Hz, to filter out spiking activity from individual neurons while preserving LFP. Throughout recording and pre-equilibration larva were flashed with two flashes of blue (488 nm) light, each lasting 100 ms and separated by a 500-ms gap, repeated every 4.5 s. This was undertaken for two purposes: to provide visual stimulation that could be detected using electrophysiology so as to ensure healthy nervous system functioning and to provide regular periodic CNS stimulation by a standard sensory pathway. Moreover, previous studies have successfully used light stimulation protocols to encourage spontaneous seizures in zebrafish expressing genes related to Dravet syndrome, thus we hypothesized it may also help to additionally sensitize animals to drug treatment (Eimon et al., 2018). Following completion of the compound exposure recording period, larval heart rate was visually assessed to confirm fish health. Additional vehicle-only experiments (identical except for no test compound exposure) were performed at regular intervals during the data acquisition phase to ensure that any changes in LFPs were not associated with the time/date of assessment.

### Data analysis: event detection

**Figure 2.**
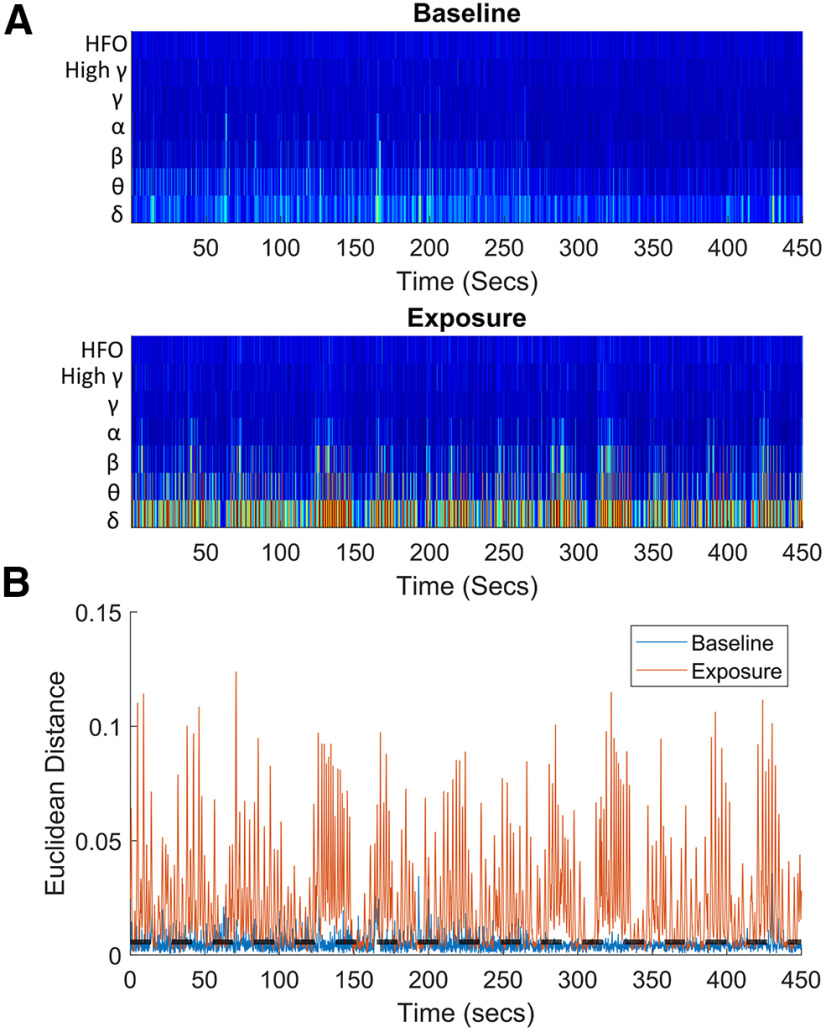
Example data obtained from *in vivo* electrophysiological recording in a 4-dpf *elavl3:GCaMP6s* zebrafish larva exposed to 62.5 μM donepezil. ***A***, A representative frequency binned wavelet transformation from a single larva, the top graph shows the baseline and the bottom wavelet transform after drug administration. These transformations were used to identify events from the full timeseries. ***B***, A plot of the Euclidean distance of each time point from the average of the baseline. The black dotted line represents the threshold for selecting events. All peaks above this line were selected and used to find events which were subsequently clustered.

The analysis described above was derived from averaging the activity over long periods. However, we also wanted to examine the presence of differences in the LFP on shorter time scales, akin to examining the morphology of epileptiform spikes or rhythm. To achieve this, we algorithmically selected shorter time periods based on differences from baseline in their frequency components, which we defined as “events.” These events were defined as periods of time 1 s long whose frequency spectra deviated significantly from the baseline. Each larva has a “drug-free” baseline period and “events” that were considered significantly different from baseline were selected. In the case of control larva this “exposure period” was also a drug free baseline period. However, because of the sensitivity of the equipment used there were still some “events” that differed enough from the baseline in the control fish to be included, although compared with the drug treated fish, these were very few in number (Fig. 4). These “erroneous” events are likely to be the result of electrical noise common in these kinds of recordings. For event detection, initially wavelet transformations were performed on the recorded voltage timeseries for both the exposure and the baseline periods to produce highly temporally resolved frequency spectra. For this a continuous 1-D wavelet transform was used, which was obtained using the analytic Morse wavelet with the symmetry parameter (γ) equal to 3, and the time-bandwidth product equal to 60, using 10 voices per octave. The frequencies of the resultant wavelets were binned into the following bands: 1–4, 4–7, 8–13, 15–30, 30–80, 80–150, and 150–500 Hz (Fig. 2*A*). These bands broadly correlate with neural frequency bands used in mammalian models (Moffett et al., 2017; Wang et al., 2020). Next, the average over time in each of these frequency bands was calculated across the entire baseline period. Subsequently the Euclidean distance between the frequency spectra of every time point in the wavelets and the average baseline frequency spectra was calculated. This allowed identification of where in the wavelet transformations deviations from the mean of the baseline occurred. After plotting the resultant Euclidean distances as shown in Figure 2*B*, we used MATLAB’s findpeaks function to identify where the Euclidean distances were greater than two standard deviations from the mean of the baseline period, selecting 1-s-long events. Next, taking all of the events selected using this first pass approach, the event wavelets for each fish were plotted separately in Euclidean space. From this, only the exposure events that were further from the centroid of the baseline events than the most distant baseline event, were selected. This process ensured we selected only the events whose spectra were distinct from those that occurred during the drug-free baseline period.

### Data analysis: wavelet of selected events

**Figure 3.**
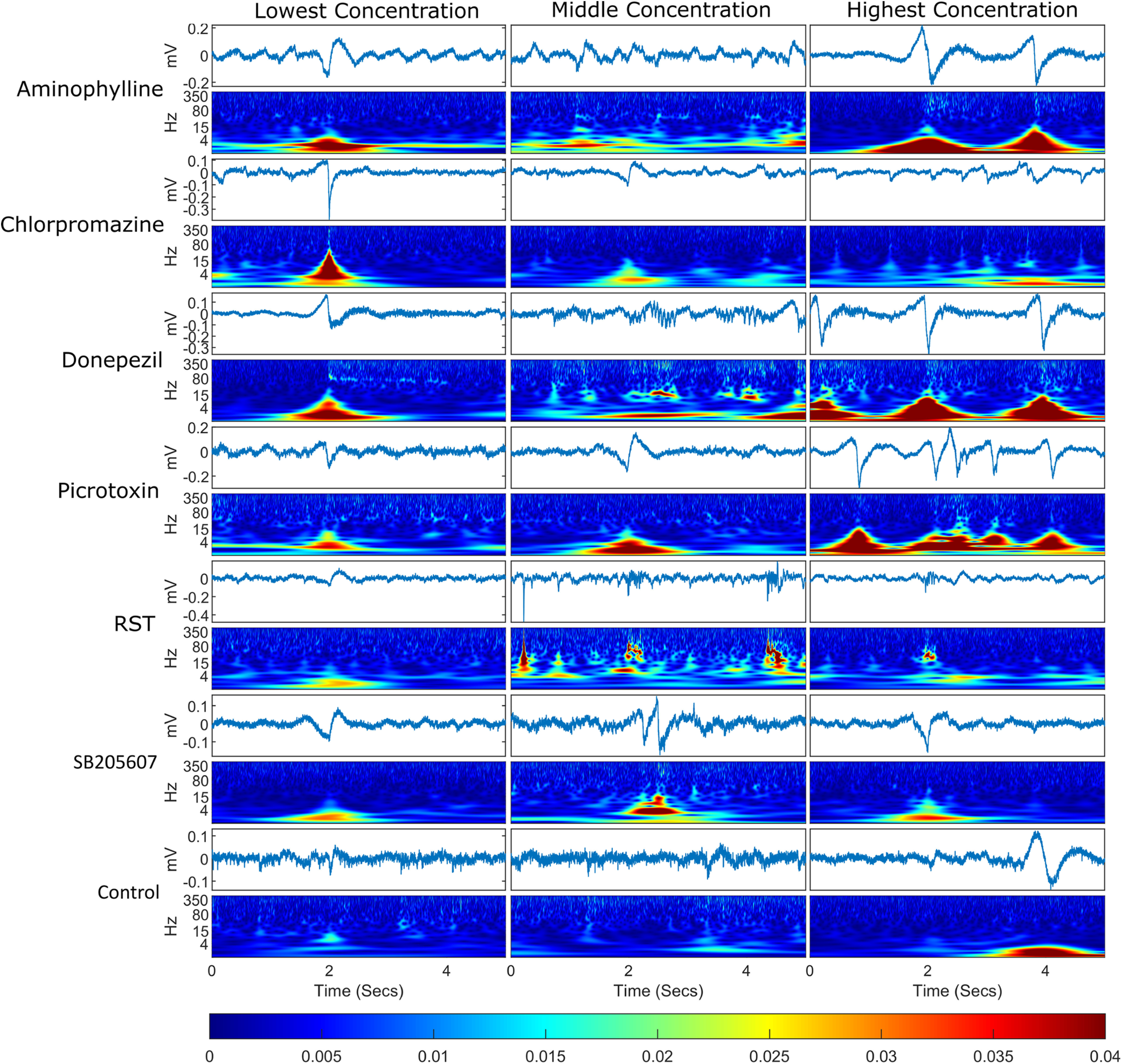
Example of an event plus 2 s either side for each treatment group. For each treatment group, the timeseries are displayed for each event with a wavelet transform below each one displaying the frequency domain over the same time period. The events selected were the events whose spectra were closest in Euclidean distance to the mean event spectra for that treatment group, meaning that these are representative of the events shown for each compound. The bottom bar shows the color scaling for the magnitude of the wavelet transformation. Each column represents a different concentration set and each row represents a different compound.

In order to better understand how the temporal profile of events differed between compounds, event epochs were selected from each treatment group of each compound. The events with the smallest Euclidean distance from the mean spectra of all the events selected for that treatment group (using the method outlined above), were selected as best representing events produced by that treatment. In addition to the presentation of the filtered timeseries, a heatmap of the wavelet transforms was also generated, as shown in Figure 3.

### Data analysis: number of selected events

**Figure 4.**
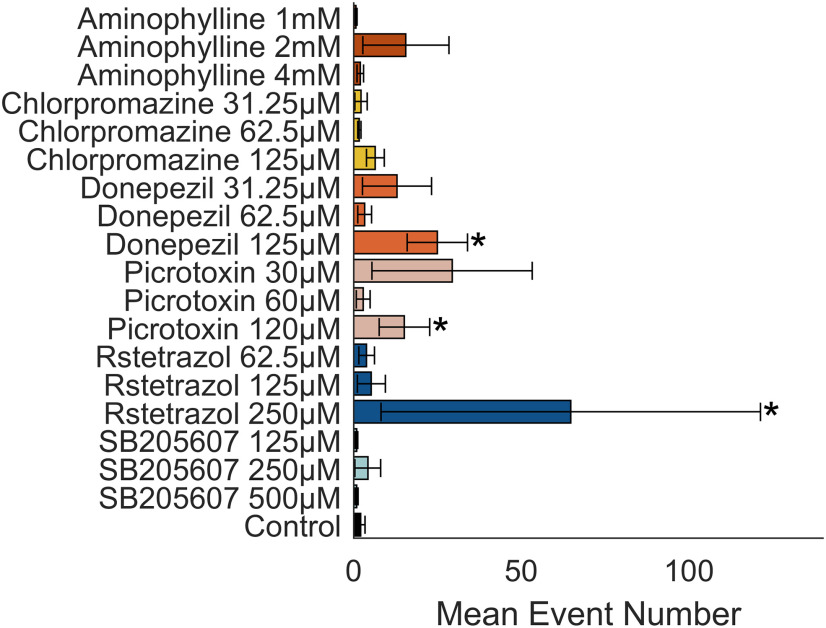
Mean number of events detected per treatment group. Bar graph showing the mean number of events per treatment group. Error bars represent the SEM (*n* = 7–8). Asterisks adjacent to the bars indicate a statistically significant difference from control (*p* < 0.05) using a Wilcoxon rank sum test corrected for multiple comparisons using the Benjamini and Hochberg method.

Moreover, the total number of detected events were calculated for each larva and compared between control and treatment groups (Fig. 4) using a Wilcoxon rank sum test, corrected for multiple comparisons using the Benjamini and Hochberg method (Benjamini and Hochberg, 1995).

### Data analysis: comparisons of area under the curve (AUC) of the LFP baseline and exposure period

**Figure 5.**
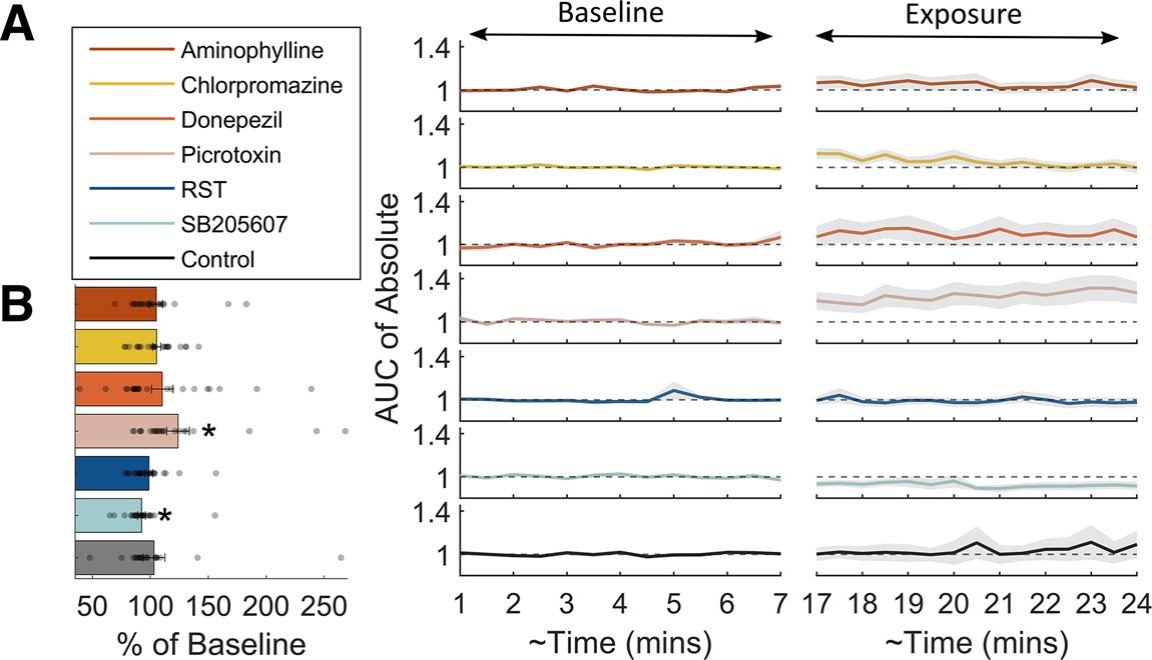
Analysis of the AUC of the Hilbert transform of the LFP recordings. ***A***, Mean baseline normalized AUC of the absolute value of the Hilbert transform of the LFP averaged into 30-s time bins. The shadows represent the SEM (*n* = 20–24). The left-hand side of the graph represents the baseline period, while the right-hand side represents the exposure period. The gap in time from 7 to 17 min is time allotted for compounds to take effect. ***B***, Shows the baseline normalized AUC for each of the compounds tested. The bars show the normalized AUC averaged across all treatment groups for each compound, while the normalized AUC of individual larvae are represented as transparent grey circles. Data are shown as the mean with error bars depicting the SEM (*n* = 20–24). An asterisk indicates a statistically significant difference between baseline and exposure periods (*p* < 0.05) using Wilcoxon signed-rank test and corrected for multiple comparisons using the Benjamini and Hochberg method (Benjamini and Hochberg, 1995).

As a first step in our analyses, we aimed to assess whether the test compounds had an effect on the overall brain activity levels, over time. Seizures are associated with excessive hypersynchronous activity, therefore fish exposed to seizurogenic drugs might be expected to display a change in overall signal amplitude. The AUC was calculated, in 30-s time bins, for the absolute value of the Hilbert transform of the filtered LFP signal to allow an assessment of the “volume” of activity over time. This allowed us to visualize the time course of oscillatory activity, whether it was increasing or decreasing throughout the exposure or baseline periods (Fig. 5*A*). Moreover, the total AUC over the baseline and exposure period was also calculated and a Wilcoxon signed-rank test (corrected for multiple comparisons using Benjamini and Hochberg method; Benjamini and Hochberg, 1995) was used to identify differences between baseline and drug-exposure recording epochs (Fig. 5*B*).

### Data analysis: spectral analysis

**Figure 6.**
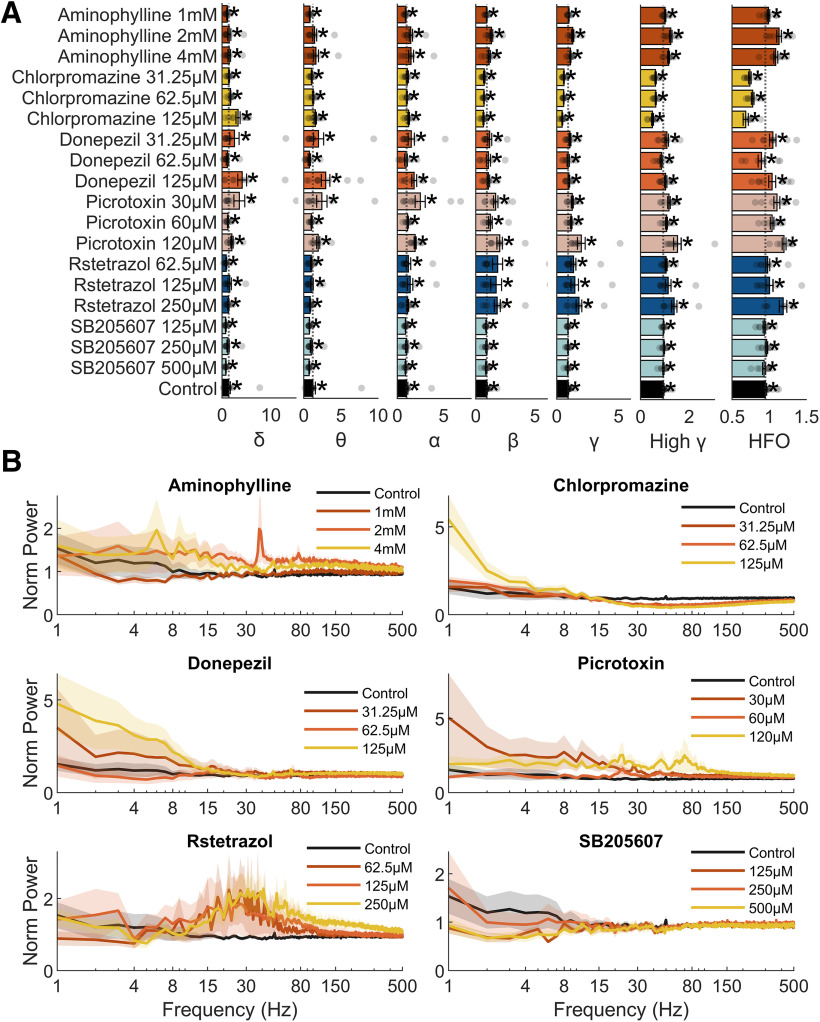
Data generated for larvae exposed to each of the test compounds after spectral analysis and categorization into specific frequency bands. ***A***, The bars show the baseline normalized mean amplitude for each of the neural frequency bands frequently used for categorizing mammalian electrophysiological data (Moffett et al., 2017; Wang et al., 2020). Data are shown as the mean with error bars showing the SEM (*n* = 7–20). The baseline normalized power for individual larvae are represented by the transparent grey circles. An asterisk indicates a statistically significant difference from control (*p* < 0.05) using Mann–Whitney *U* tests and corrected for multiple comparisons using the Benjamini and Hochberg method (Benjamini and Hochberg, 1995). ***B***, Mean baseline normalized power spectra for each compound treatment group. In this case the *x*-axis is scaled to the common logarithm. Shading represents the SEM for each data point (*n* = 7–20) across the power spectra. Black lines here indicate the mean of the control animal power spectra and therefore are the same for each graph.

Among the most commonly used approaches for assessing changes in neural activity patterns is frequency domain analysis, which allows investigation into the contribution of oscillations at different frequencies to the overall signal. Commonly in human EEG or mammalian animal model LFP datasets, oscillations are binned into bands of behavioral relevance, and changes in the power of these bands can correlate with transitions in behavioral or cognitive state (Başar et al., 2001). While the same frequency bands may not be as behaviorally-relevant in zebrafish, we hypothesized that because of the similar underlying neurophysiology of mammals and zebrafish, interpreting spectral changes in the context of the same bands may provide useful insights into underlying mechanisms of seizurogenesis. Furthermore, performing such analysis in the 4-dpf larval zebrafish served to provide a shared context which, it was hypothesized, would be important for making interspecies comparisons of electrophysiological responses to seizurogenic chemical treatment. Fourier transforms were performed on the signal from baseline and exposure periods of each fish, and the resulting spectra during each exposure period were normalized to the corresponding spectra of the baseline recordings. Next, the mean normalized spectra for each fish were averaged within treatment groups (Fig. 6*B*). Finally, normalized spectra were binned into frequency bands used for studying mammalian and human EEG data (Moffett et al., 2017; Wang et al., 2020). These bands were: δ (1–4 Hz), θ (4–7 Hz), α/μ (8–13 Hz), β (15–30 Hz), γ (30–80 Hz) and high γ (80–150 Hz), and high frequency oscillations (HFO) (150–500 Hz). The activity in each band was then compared between test compound-treated and control fish using Mann–Whitney *U* tests corrected for multiple comparisons using the Benjamini and Hochberg method (Benjamini and Hochberg, 1995; Fig. 6*A*; Table 2).

**Table 2:** All statistical test outputs from frequency band analyses

Frequency band	Compound	Concentration	*P*-value	Corrected *p*-value	Test statistic	*Z*-value
δ	Chlorpromazine	62.5 μm	0.013812	0.03942	235	−2.46211
δ	Chlorpromazine	125 μm	0.010224	0.035258	239	−2.56817
δ	Picrotoxin	120 μm	0.013812	0.038106	235	−2.46211
θ	Picrotoxin	120 μm	0.002566	0.013276	225	−3.01539
α	Picrotoxin	120 μm	0.000544	0.003464	217	−3.45802
β	Aminophylline	2 mm	0.010089	0.036306	233	−2.57277
β	Chlorpromazine	31.25 μm	0.011825	0.037643	340	2.517311
β	Chlorpromazine	62.5 μm	0.00438	0.018126	332	2.849409
β	Chlorpromazine	125 μm	0.011825	0.036249	340	2.517311
β	Picrotoxin	120 μm	0.000442	0.003329	216	−3.51335
γ	Aminophylline	2 mm	0.002566	0.012495	225	−3.01539
γ	Chlorpromazine	31.25 μm	0.0001	0.002071	367	3.890389
γ	Chlorpromazine	62.5 μm	0.000188	0.002223	348	3.734663
γ	Chlorpromazine	125 μm	0.000053	0.004369	370	4.042954
γ	Picrotoxin	120 μm	0.00029	0.002668	214	−3.62401
γ	RST	250 μm	0.011825	0.034954	240	−2.51731
High γ	Aminophylline	2 mm	0.002566	0.011801	225	−3.01539
High γ	Aminophylline	4 mm	0.011821	0.039135	234	−2.51744
High γ	Chlorpromazine	31.25 μm	0.000081	0.002236	368	3.941244
High γ	Chlorpromazine	62.5 μm	0.000188	0.001945	348	3.734663
High γ	Chlorpromazine	125 μm	0.000053	0.002184	370	4.042954
High γ	Picrotoxin	120 μm	0.000442	0.003052	216	−3.51335
High γ	RST	250 μm	0.00293	0.012763	231	−2.975
HFO	Aminophylline	2 mm	0.001466	0.008088	222	−3.18138
HFO	Aminophylline	4 mm	0.008586	0.032303	232	−2.6281
HFO	Chlorpromazine	31.25 μm	0.0001	0.001657	367	3.890389
HFO	Chlorpromazine	62.5 μm	0.000995	0.00588	340	3.292036
HFO	Chlorpromazine	125 μm	0.000123	0.0017	366	3.839535
HFO	Picrotoxin	120 μm	0.00029	0.002401	214	−3.62401
HFO	RST	250 μm	0.004766	0.018784	234	−2.82244

Activity in each frequency band compared between test compound-treated and control fish using unpaired Wilcoxon rank sum test and corrected for multiple comparisons using the Benjamini and Hochberg method (Benjamini and Hochberg, 1995). The columns from left to right contain the relevant frequency band, compound, concentration of exposure, *p*-value, Benjamini and Hochberg corrected *p*-value, rank-sum test statistic, and corresponding z-statistic computed when the method is “approximate.” All statistics were undertaken using the MATLAB statistics and machine learning toolbox (MATLAB ranksum - MathWorks United Kingdom; Wilcoxon rank sum test, 2021). HFO, high frequency oscillation.

### Data analysis: classical multidimensional scaling (MDS) of baseline normalized spectral data

In order to visualize the similarity or differences between representative spectra for each treatment group, classical multidimensional scaling was performed across the average normalized power spectra for each fish. Subsequently, the mean of the first two coordinates was calculated across treatments groups, and were plotted. The eigenvalues for these coordinates accounted for >74% of the sum of all the eigenvalues.

### Code accessibility

The code/software described in the paper is freely available online at: https://github.com/jp908/Ephys-Seizure-ZF.

## Results

### Mean event and wavelet analysis

The results of the mean event and wavelet analysis are summarized in Figure 3. Exposure to the compounds tested resulted in bursts of rhythmic activity of specific frequencies consistent with changes in the spectra observed in the previous analyses (Fig. 6). Chlorpromazine’s wavelet spectrograms, for example, showed a distinct amelioration of >80-Hz activity particularly in the higher concentrations, in addition to bursts of θ, δ, and α range activity. The events identified in animals exposed to donepezil were categorized by a gradual polarization followed by a large low-frequency event in the <15-Hz range with an additional high-frequency component at the peak of, and subsequent to, the main depolarization. However, there was also evidence of bursting type high-frequency activity. Picrotoxin exposure resulted in wavelet spectrograms characterized by a high-amplitude activity in the 80 Hz or less frequency range, and appeared to show a distinct dose-dependent increase in activity. RST exposed animals were observed to contain a bursting type of high-frequency activity in addition to sharp spike-type discharges, as illustrated most clearly at the middle concentration. Notably RST appeared to lack much of the lower frequency component. Both aminophylline and SB205607 appeared to induce medium sized events, although notably the highest concentration of aminophylline appeared to have some similarities with donepezil’s high concentration event profile.

### Number of detected events

Exposure to aminophylline, chlorpromazine, donepezil, and RST appeared to increase the number of events detected in a dose-dependent manner (Fig. 4). The highest concentrations of, donepezil (*p* = 0.0354 *z*-value = −2.764, test statistic = 239.5), picrotoxin (*p* = 0.0273, *z*-value = −2.715, test statistic = 234), and RST (*p* = 0.0159, *z*-value = –3.220, test statistic = 229.5) resulted in a significant increase in the number of detected events as compared with the control.

### Changes in AUC between baseline and exposure

The results of the changes in AUC from baseline to exposure are summarized in Figure 5. Only picrotoxin (*p* = 0.0081, corrected-*p* = 0.0104, *W* = 51, *z*-value = −2.6461) and SB205607 (*p* = 0.0006, corrected-*p* = 0.0015, *W* = 270, *z*-value = 3.4286) exposure resulted in significant changes in AUC between the baseline and exposure periods, exhibiting increased and decreased AUC, respectively. The AUC of picrotoxin appeared to oscillate slightly across 30-s time bins with a slight upward gradient.

### Spectral frequency band analysis

The results of the spectral analysis of the *in vivo* LFP data are summarized in Figure 6 and Table 2. From these data, it can be seen that exposure to the highest concentrations of chlorpromazine and picrotoxin resulted in a significant increase in activity in the slower δ-frequency band compared with the control animals. In the higher frequency γ, high γ, and HFO bands, significant increases in activity were observed after exposure to (RS)-(tetrazol-5-yl)glycine (RST), picrotoxin and aminophylline. Interestingly, exposure to chlorpromazine resulted in significantly reduced, β, γ, high γ, and HFO band activity at all of the concentrations tested, and this was not observed for any other compounds assessed here. Exposure to SB205607 and donepezil resulted in no significant changes in any of the frequency bands measured. In addition, exposure to picrotoxin resulted in increases in α band oscillatory activity, while aminophylline and picrotoxin exposure resulted in increases in β power. Notably, picrotoxin resulted in an increase in power in every single frequency band, but only at the highest concentration.

### Classical MDS

**Figure 7.**
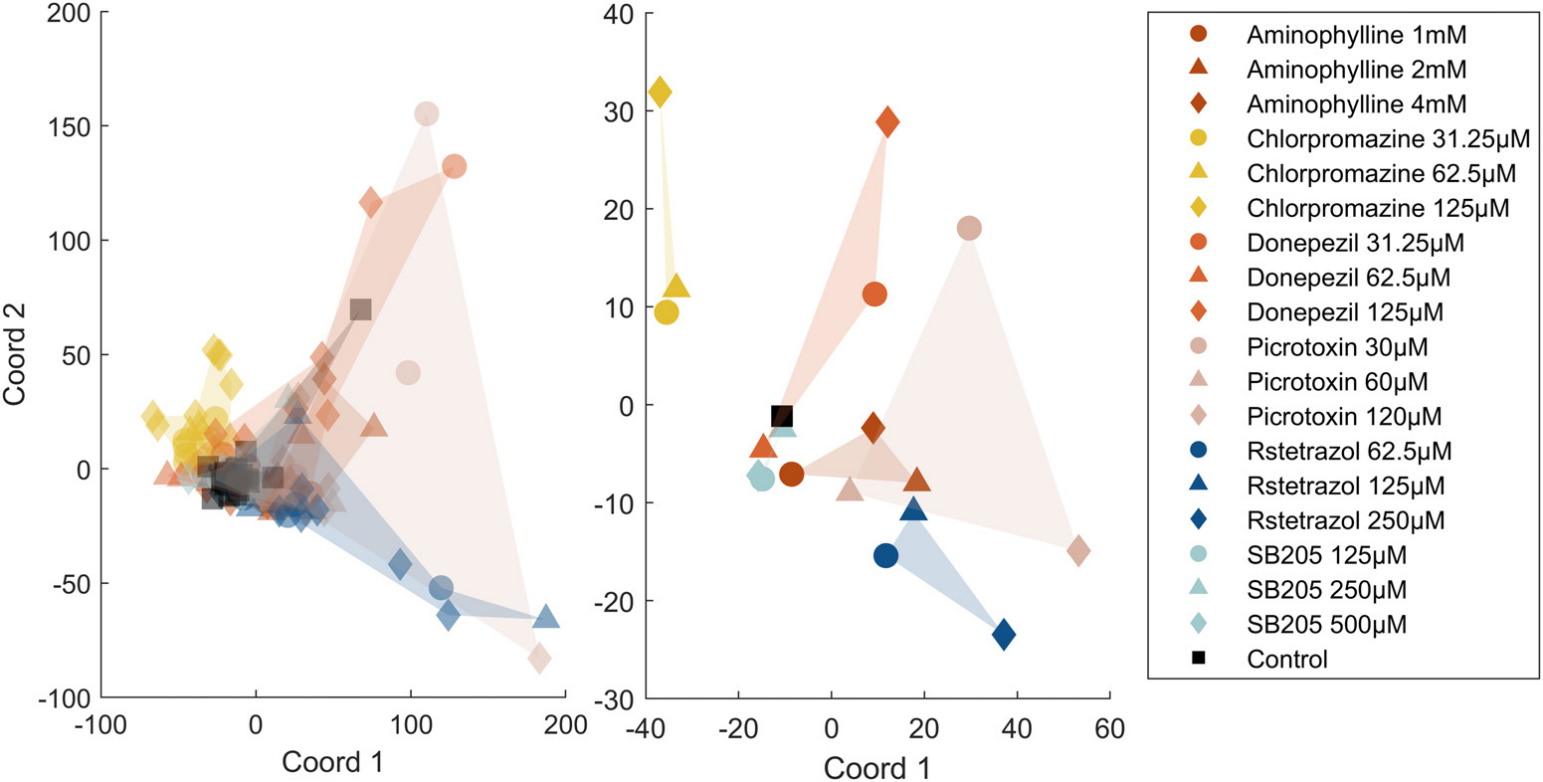
Multi-dimensional scaling (MDS) of the normalized power spectra. Left-hand image, A scatter graph of the first two coordinates produced after classical MDS of each individual larva. In both plots, the circles represent the lowest concentration, the triangles the middle concentration, and the diamonds the highest concentration used for each treatment group. The black square represents the control group. Right-hand image, A scatter graph of the first two coordinates produced after classical MDS of the mean normalized power spectra of each treatment group (see Fig. 6*B*).

Classical MDS of the spectral data revealed the distribution of each treatment group in two dimensions. The outer regions of the resultant scatter plot (Fig. 7) are occupied by the highest concentration of chlorpromazine, donepezil, RST, and picrotoxin. Notably there was overlap of the middle concentration of aminophylline and picrotoxin, likely because of similar increases in β, γ, and high-frequency oscillations. SB205607 and the lowest concentration of aminophylline lie closest to the control reflecting the relative absence of induced effects on the parameters measured. The highest concentration of chlorpromazine is located furthest from RST and picrotoxin likely reflecting its reduction in oscillations at >15 Hz. Furthermore, RST and picrotoxin are located on the right-hand side of the scatterplot likely because of their notable induced increases in the magnitude of oscillations at >15 Hz.

## Discussion

Using *in vivo* mid-brain LFP recordings in 4-dpf larval zebrafish, we demonstrate responsiveness to a range of pharmacological agents implicated in the induction of seizures in mammals. The data we present show that exposure to chlorpromazine (a phenothiazine antipsychotic), donepezil (an acetylcholinesterase inhibitor), picrotoxin (a GABA_A_R antagonist) and RST (an NMDAR agonist) results in quantifiable, concentration-dependent, altered neuronal electrophysiology. Furthermore, the electrographic responses exhibited differed between each drug, and in some cases exhibited characteristics commensurate with the induction of interictal and ictal electrophysiology phenotypes similar to those classified in humans (Fisher et al., 2014b).

### Electrographic phenotypes of representative traces

Although seizure phenotypes from electrographic recordings are extremely diverse, the main aim of our study was to explore what “abnormal” LFP recordings might emerge following exposure of zebrafish larvae to drugs acting via known seizurogenic mechanisms. Furthermore, from these phenotypes, we aimed to classify such patterns in the context of their frequency components, and to compare these components to human EEG data and models of seizure in mammals. To this end we compared the representative traces that were selected using our event detection algorithm with specific examples of preictal, interictal, and ictal type waveforms, as reported previously (Fisher et al., 2014b). The representative trace for the highest concentration of chlorpromazine, for example, appeared to comprise of rhythmical evolving θ, δ, and α frequencies, which is an electrophysiological phenotype commonly seen preceding seizure in EEG traces recorded clinically (Fisher et al., 2014b). Similarly, the high-amplitude, high-frequency, discharges observed in the representative traces for the intermediate exposure concentrations for both donepezil and RST share similarities with intracerebral stereo-EEG recordings of seizures reported in mammals (Fisher et al., 2014b). These discharges also contained very high-frequency (80–500 Hz) components similar to examples of seizures reported previously in mammals (Mackenzie et al., 2002; Jiruska et al., 2013, 2017; Shiri et al., 2015; Gliske et al., 2017; Wang et al., 2020). The representative traces for the highest concentration of aminophylline, in addition to that seen for the lowest and highest concentrations of donepezil, appear consistent with electrodecromental EEG patterns, a electrophysiological phenotype commonly seen preceding seizure in EEG traces recorded clinically (Fisher et al., 2014b).

Qualitative comparisons of representative traces for the highest concentration of aminophylline, donepezil, and picrotoxin in our study, with traces of spontaneous ictal and interictal activity identified by (Griffin et al., 2021) in their recording of LFPs in zebrafish (where they were screening for potentially seizurogenic genetic modifications), showed strong similarities. Moreover, the traces for the medium and high concentrations of RST appeared similar to their definition of an ictal-type waveform. These similarities support a good degree of consistency between drug induced seizures and spontaneous seizures, although a more extensive comparison would be necessary to confirm this. Compared with behavioral and imaging-based assessments of seizures in zebrafish (Winter et al., 2008; Alfaro et al., 2011; Afrikanova et al., 2013; Baraban et al., 2013; Winter et al., 2017; Burgstaller et al., 2019; Winter et al., 2021), *in vivo* LFP recordings provide a direct measure of the electrographic response of model organisms to neuroactive drug treatment. This is important as the data generated more readily allows comparisons between model species (Table 3), a feature which is crucial for evaluating the translational power of the zebrafish as a surrogate for understanding the effects of neuroactive drugs in higher vertebrates, including humans.

**Table 3:** Summary of information comparing the effect of selected drugs on EEG in mammals from published data with the data obtained for zebrafish in this study

Compound	Effects observed in 4-dpf zebrafish LFP recordings undertaken in this study	Effects reported from EEG recordings undertaken in mammals
Aminophylline	Increase in the amount of β and γ oscillations (Fig. 6) and the presence of electrodecromental type preictal discharges (Fig. 3).	Little frequency domain analysis of EEG exists for aminophylline; however, visual assessment of EEG traces from rats exposed to high doses of aminophylline show epileptiform discharges of high amplitude and frequency (Chu, 1981).
Chlorpromazine (antipsychotic)	We observed a significant reduction in all of the frequency bands >15 Hz on exposure to chlorpromazine (Fig. 6).	Antipsychotic medications haloperidol and clozapine significantly reduce γ power (Olszewski et al., 2013; Sun et al., 2020).
Donepezil	Apparent increase in theta oscillations (the largest mean difference of all the compounds) although it did not reach statistical significance (Fig. 6). Events with a large θ component (Fig. 3).	Rats exposed to Donepezil show increases in cortical EEG oscillations in the θ frequency band (Ahnaou et al., 2014).
Picrotoxin	Significant increases in gamma, fast γ activity, and high-frequency oscillation amplitude (Fig. 6). This is consistent with studies showing high-frequency bursting type LFPs (Baraban, 2013).	Picrotoxin increases γ oscillations in the olfactory bulb, an effect that could be suppressed via inhibition of ionotropic glutamate receptors (Lepousez and Lledo, 2013). Mouse brain slices show induction of HFOs via picrotoxin exposure (Shiri et al., 2015).
RST	Significant increases in γ and fast γ activity (Fig. 6).	In rats, NMDA agonists induce acetylcholine release and affect an increase in both beta and γ oscillations in the basal forebrain (Fournier et al., 2004).
SB205607	Few effects of SB205607 on LFP in zebrafish.	No studies found for the electrophysiology of SB205607 in mammals.

### Trace amplitude and seizure

Given that seizures are considered to be high-amplitude events, measuring the change in the average AUC induced by the different compounds was initially undertaken. Notably the largest increase in AUC was observed after exposure to picrotoxin which is commonly used in animal models as a seizure precipitant (Winter et al., 2008; Baraban, 2013; Ridler et al., 2018; Fan et al., 2019). This is consistent with previous studies in zebrafish exposed to picrotoxin that reported the presence of high-amplitude events (Baraban, 2013). Interestingly, however, the NMDA-receptor agonist RST, which is also a well-known model convulsant, induced no change in AUC despite inducing changes in other measured parameters. It is worth noting that seizure like states are often characterized by periods of bursting activity with intermittent quiescence (Barnett et al., 2013). In this context, it is possible that periods of seizure like activity induced via RST are bursting in nature, with long intervals in-between and thus not best detected using our protocol. In support of this, on a more detailed analysis of the spectral components of its electrographic signature, the resultant data suggested that RST exposure was associated with elevated neural activity in specific frequency-bands, rather than showing a more generalized elevated activity. In a previous study using Ca^2+^ imaging (Winter et al., 2021), it was also reported that there was a relative absence of oscillatory activity after exposure to RST (and the pharmacologically similar NMDA). Given that here we saw elevated activity only in the >15-Hz frequency range (see below), and notwithstanding the sensitivity of Ca^2+^ dye-based imaging, it is possible that in the previous study, the temporal resolution of the imaging-based approach used was insufficient to capture such rapid oscillations thus highlighting the relative advantages and disadvantages of each type of approach when studying drug-induced neuronal events.

### Neural oscillations zebrafish versus mammals

The binning of generated frequency spectra into specific frequency bands is a commonly used approach in the analysis of mammalian electrophysiological data (Başar et al., 2001). We adopted this approach primarily to directly compare neural activity signatures between 4-dpf larval zebrafish and mammalian models of drug-induced seizures. It should be noted that we consider the changes seen in the different frequency bands for all drugs to be as a result of bursts of rhythmic activity. This means there are both periodic and aperiodic contributions to the frequency spectra. We believe this is the case, because the event detection algorithm utilized here identified bursts of rhythmic activity whose spectra were consistent with the frequency spectra performed on the full recordings. Moreover, the number of these events was not adequate to constitute the full traces. As such our analyses suggested that drug exposure resulted in transient bursts of rhythmic activity that altered the overall spectral profile of the recordings, as opposed to sustained oscillatory activity.

In multiple EEG studies undertaken in mice, the antipsychotic medications haloperidol and clozapine have been shown to significantly reduce γ power (Olszewski et al., 2013; Sun et al., 2020). Here, we also observed a significant reduction in all of the frequency bands of >15Hz on exposure to chlorpromazine. Both chlorpromazine and haloperidol broadly reduce monoamine signaling, a neurotransmitter subgroup that is highly conserved between larval zebrafish and mammals in both their molecular underpinnings and behavioral functionality (Maximino et al., 2016). Indeed, haloperidol has been shown to induce locomotor impairments in zebrafish, an effect mirrored in the common side effects in humans (e.g., drowsiness, dizziness, and neuromuscular dysfunction; Giacomini et al., 2006). Chlorpromazine has also been shown to induce significant alterations in 4-dpf larval brain functional connectivity (Winter et al., 2021), and our data here also show comparable oscillatory activity induced by this antipsychotic compound between zebrafish and mammals. The confirmation here of shared brain oscillatory changes, in addition to the aforementioned similar behavioral manifestations, suggests that the established links between neural frequency bands and mammalian behavior may have some translatability to zebrafish.

Exposure to the acetylcholinesterase inhibitor donepezil increased power in the θ frequency bands in larval zebrafish. In mammals, θ and γ oscillations are associated with successful memory recall (Barnett et al., 2013). Notably, a mechanistically similar compound and cholinesterase inhibitor, physostigmine, has been shown to reverse scopolamine-induced learning impairment in zebrafish, suggesting effectiveness in reversing impaired cognition (Kim et al., 2010a). In this context it would be interesting to assess the behavioral response of zebrafish to donepezil specifically given the apparent enhancement of θ oscillations in the electrophysiology data we present here. Indeed, observation of donepezil-induced cognitive enhancement in larval zebrafish would provide further evidence of a similar relationship between brain oscillatory patterns and behavioral manifestations in larval zebrafish as those observed in mammals.

Perturbations in oscillations in the γ and high γ ranges are common across a variety of psychiatric disorders, including attention deficit hyperactivity disorder, schizophrenia and, of particular relevance here, epilepsy (Herrmann and Demiralp, 2005). Epilepsy specifically is associated with an increase in γ oscillations likely because of cortical excitation (Herrmann and Demiralp, 2005). Indeed, in human EEGs, oscillations in the γ and fast γ range precede interictal epileptiform spikes in the seizure onset zone (Jiruska et al., 2013). Similarly, in the nucleus accumbens of rats, there are increases in γ power when seizure kindling is induced using kainic acid (Ma and Leung, 2010). Here, we observed significant increases in γ and fast γ activity after exposure to the two seizure precipitants picrotoxin and RST, in addition to aminophylline. Previously published work in mice has shown that treatment with picrotoxin has been shown to increase γ oscillations in the olfactory bulb, an effect that could be suppressed via inhibition of ionotropic glutamate receptors (Lepousez and Lledo, 2013). In addition, in rats, NMDA agonists induce acetylcholine release and affect an increase in both β and γ oscillations in the basal forebrain (Fournier et al., 2004). In our study, both picrotoxin and RST appeared to induce abnormal electrographic events, similar to those recorded in previous studies in zebrafish (Baraban et al., 2005; Hortopan et al., 2010; Afrikanova et al., 2013; Löscher, 2017; Copmans et al., 2019) and consistent with seizures observed in human EEG (Fisher et al., 2014b). Notably our data suggest that these events, much like in humans and rats, were characterized by an increase in γ and high-γ frequency oscillations. The fact that chlorpromazine appeared to uniquely reduce γ and high γ oscillations is interesting and raises the issue of primary versus secondary pharmacological activity and dosing levels. Certainly, chlorpromazine is known to have potent sedative effects linked to its well-known H_1_ activity (von Coburg et al., 2009) and is typically associated with a seizure-threshold lowering effect rather than a direct seizurogenic effect as measured using our exposure method (Chi et al., 2017).

The highest frequency band we measured, in the range between 150 and 500 Hz, contained HFOs (Wang et al., 2020). HFOs are strongly correlated with the epileptogenic zone and have been implicated as a useful biomarker of epilepsy (Lévesque and Avoli, 2019), although their use to prospectively define the epileptogenic zone is not clear (Jacobs et al., 2018). Indeed, fast ripples (250–500 Hz) appear to be highly indicative of epileptic tissue in both human conditions and animal models (Jiruska et al., 2017). HFOs are believed to occur as a result of the synchronization of fast firing within populations of interconnected neurons generating high-frequency population spikes which, when recorded extracellularly, present as an HFO event (Jiruska et al., 2017). Individual Pyramidal neurons cannot fire fast enough to account for oscillations higher than 300 Hz, thus, fast ripple oscillations have been proposed to be generated via the action of synchronized, but out-of-phase, neurons (Jiruska et al., 2013). Here, a significant increase in the magnitude of HFOs was observed after exposure to aminophylline, picrotoxin, and RST, while chlorpromazine appeared to reduce the magnitude of HFOs. This is consistent with studies in mouse brain slices showing induction of HFOs via picrotoxin exposure (Shiri et al., 2015). Moreover, automated event detection designed to categorize events unique from baseline identified several events across these compounds that contained HFOs, including after exposure to donepezil, RST, and picrotoxin across all concentrations.

### Study limitations

Exposure to the δ-opioid agonist, SB205607, showed little evidence of altered brain electrophysiology despite showing significantly elevated activity in a number of brain regions at 500 μm (the top concentration used here) in an imaging-based assessment undertaken in 4-dpf zebrafish (Winter et al., 2021). Other studies have reported insensitivity of the larval zebrafish to specific δOR agonists such as SNC80 (Rodriguez and Gonzalez-Nunez, 2006) although this does not explain why previous work with SB205607 suggested some degree of neuroactivity (Winter et al., 2021). One possible reason for this could be explained by the relative strengths and weaknesses of electrophysiological versus imaging-based neural functional assessments. Electrophysiological assessment offers unparalleled levels of temporal resolution, whereas imaging-based approach offer much greater spatial coverage. Given the LFP approach used here measures electrical activity in a small population of neurons in one part of the brain it is possible that this region was not activated by SB205607 exposure. Certainly, this would be supported by the data from our Ca^2+^ imaging work (Winter et al., 2021), which suggested comparatively low levels of activity in mid brain regions compared with the hind brain. This could be investigated further by the use of multielectrode arrays to allow for greater spatial coverage during LFP recordings (Hong et al., 2016).

Despite the existence of notable similarities between the LFPs recorded in zebrafish and mammalian electrophysiology data, there are limitations to the larval zebrafish as a model. Specifically, *in vitro* LFP recordings taken in mammals are often performed on specific anatomic structures which in the case of seizure and epilepsy research are recognized as hyperexcitatory in nature, such as the hippocampus. In zebrafish larvae, recording from specific brain structures is extremely challenging due the small size of the brain, thus recordings are generally taken from the midbrain or the forebrain with little ability to differentiate between specific sub regions of higher or lower relevance. In this respect, the use of genetically modified models in which specific neural circuits or populations are labeled may prove extremely useful and allow more precise placement of electrodes in structures that have been identified as especially appropriate for measuring the type of brain activity being investigated.

In summary, *in vivo* LFP-mediated assessment of neural activity in 4-dpf zebrafish larvae revealed responsiveness to seizurogenic compounds that act via a range of pharmacological mechanisms of action. Furthermore, the resultant electrographic profiles exhibited by 4-dpf zebrafish larvae exposed to a number of these compounds show clear differences in their characteristics and in some cases share notable similarities with the signatures exhibited by mammalian, including human, electrophysiological profiles. The data generated here add to the body of data supporting the use of the larval zebrafish as a complimentary and potentially alternative model for the study of seizures and epilepsy and also provide further insight into the electrophysiological characteristics of seizures generated in nonmammalian species.

## Synthesis

Reviewing Editor: Katalin Toth, University of Ottawa Decisions are customarily a result of the Reviewing Editor and the peer reviewers coming together and discussing their recommendations until a consensus is reached. When revisions are invited, a fact-based synthesis statement explaining their decision and outlining what is needed to prepare a revision will be listed below. The following reviewer(s) agreed to reveal their identity: Richard Rosch.

The reviewers found the data useful, the zebrafish model here has clear advantages, as a direct comparison of drug effect is possible here. Both reviewers suggest several important changes to improve the manuscript. The most significant changes are from Rev. #2. The authors would need to make it clear how they defined ictal and interictal events, elaborate on cross-species comparison, and better define oscillations. In addition, they also should address all the other smaller issues raised by the reviewers.
