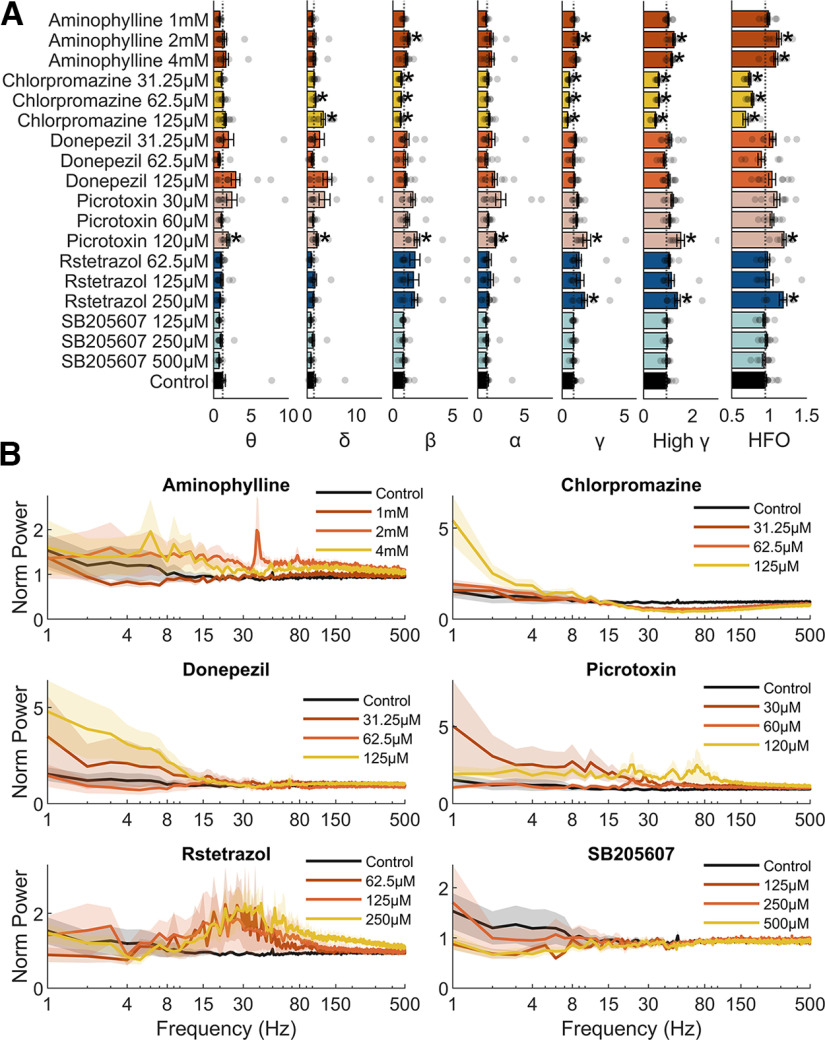# Erratum: Pinion et al., “Differential Electrographic Signatures Generated by Mechanistically-Diverse Seizurogenic Compounds in the Larval Zebrafish Brain”

**DOI:** 10.1523/ENEURO.0291-22.2022

**Published:** 2022-08-02

In the article “Differential Electrographic Signatures Generated by Mechanistically-Diverse Seizurogenic Compounds in the Larval Zebrafish Brain,” by Joseph Pinion, Callum Walsh, Marc Goodfellow, Andrew D. Randall, Charles R. Tyler, and Matthew J. Winter, which was published online on February 28, 2022, Figure 6 appeared incorrectly. The asterisks that indicate statistical significance were inadvertently applied across all treatment groups because of a bug in the code for the figure. The revised figure appears below, and the online version has been corrected.

**Figure 6.**